# Cervical masses as manifestation of papillary thyroid carcinomas ≤10 mm in diameter, in patients with unknown thyroid disease

**DOI:** 10.1186/1756-6614-1-8

**Published:** 2008-12-06

**Authors:** Kalliopi Pazaitou-Panayiotou, Maria Alevizaki, Maria Boudina, Apostolos Drimonitis, Anastasia Kiziridou, Iraklis Vainas

**Affiliations:** 1 Department of Endocrinology-Endocrine Oncology, Theagenion Cancer Hospital, Thessaloniki, Greece; 2 Endocrine Unit, Evgenideion Hospital and Dept Medical Therapeutics, Alexandra Hospital, Athens University School of Medicine, Athens, Greece; 3 Department of Pathology, Theagenion Cancer Hospital, Thessaloniki, Greece

## Abstract

**Background:**

Papillary thyroid microcarcinomas are tumors often found accidentally after thyroidectomy for other thyroid disorders.

**Methods:**

Patients with enlarged lateral cervical masses, with unknown thyroid disease, found to have metastases from papillary thyroid carcinoma ≤10 mm in diameter, were compared to patients operated on for nodular or multinodular goiter, who were incidentally found to have papillary thyroid microcarcinomas.

**Results:**

Group A included 24 patients with an enlarged lateral cervical mass whereas group B included 30 patients presenting with nodular or multinodular goiter. Patients in both groups underwent surgery. After thyroidectomy and lymph node dissection, pathology revealed multifocal papillary carcinomas of 1–10 mm, with invasion of the thyroid capsule and surrounding soft tissue in most of the cases in group A. Two patients presented with distant metastases at diagnosis which were surgically removed. During follow up, 3 patients (12.5%) presented with new cervical metastases which were surgically removed or treated with additional radioactive iodine. At last follow-up, all patients were alive. In contrast, all patients in group B had unifocal papillary thyroid carcinoma 1–10 mm in maximum diameter, with no infiltration or extension into the adjacent tissue, or cervical lymph node metastases.

**Conclusion:**

Two groups of papillary thyroid microcarcinomas characterized by different clinical and biological behaviours are identified. Significant differences were found between these groups concerning the age, tumor size, number of tumor foci, lymph nodes metastases and extrathyroidal extension of the tumor. Papillary thyroid carcinomas of small (≤10 mm) size may have aggressive behaviour or be metastatic, and this subgroup should be treated and followed up as are other large, differentiated thyroid cancers.

## Background

Papillary microcarcinomas of the thyroid are defined as tumors 10 mm or less in diameter [1,2] which are often not detectable upon clinical examination, but are frequently found accidentally [3] after thyroidectomy for other thyroid disorders. Their behaviour is benign most of the time. Many are not diagnosed during the patient's lifetime. It has been reported that occult papillary carcinomas may be found at autopsy in up to 24% of the cases [4,5]. They are considered to be a subgroup of well-differentiated carcinomas of the thyroid and it was believed that they could rarely cause cervical or distant metastases. However papillary microcarcinomas may present in a more aggressive manner with lymph nodes or even distant metastases [6,7] and, in many reports on this problem, lymph node metastases or extrathyroidal extension were found in up to 40.3% of the cases [8-12]

Patients with nodular or multinodular goiters may have papillary thyroid microcar-cinomas that are found incidentally after thyroidectomy. We compared results from consecutive cases of thyroidectomies which were performed to remove goiter where small carcinomas were found incidentally after surgery to cases of carcinomas of the thyroid ≤10 mm in diameter diagnosed after they had metastasized to cervical lymph nodes in patients with unknown thyroid disease.

## Patients and methods

Twenty-four patients including 19 women and 5 men aged 16–63 years, (mean age 39.96 ± 13.91 years) were referred to the internal medicine or surgical outpatients' clinic at Theagenion Cancer Hospital from 1993–2005 because of a neck mass that was painless (group A). Of these masses, 15 were on the right, and 9 on the left side; they measured 3–10 cm in maximum diameter. Clinical examination revealed the existence of one or more cervical lymph nodes in all patients and absence of goitre. Thyroid function tests were normal. No patient had previously received head and neck irradiation therapy, or had a known history of thyroid disease.

Inflammatory diseases that can cause cervical lymphadenopathy were excluded in all patients. An ultrasound scan of the lateral cervical mass revealed 2 large cystic formations with few solid elements in 2 patients, which were considered to be bran-chial cysts. In 22 patients, multiple enlarged solid hypoechoic masses, some with areas of fusion were observed, which probably represented lymph nodes.

Fine needle aspiration (FNA) followed by cytological examination of the aspirate was performed in 5 patients, while 19 patients had a lymph node dissection followed by histological examination of the node. In all patients, cytology or pathology revealed metastases from papillary thyroid carcinoma, a finding that led to the decision for total thyroidectomy and lymph node dissection. As the original examination was performed in the non-specialized clinic, ultrasound scan of the thyroid was pre-operatively performed in only 17 patients, after the diagnosis of metastatic papillary thyroid carcinoma was established; this revealed a single small hypoechoic nodule in 8 patients and 2 nodules in 4 patients, all sized 4–10 mm in diameter, and increased intranodular vascularization, one hyperechoic nodule 10 mm in diameter in 2 patients, and normal findings in 3 patients. Unfortunately no preoperative thyroid US was performed in the remaining 7 patients. In addition, 16 of the 24 patients, underwent thyroid ^99^Tc scintigraphy which showed a small cold nodule in 4, increased uptake of radionucleotide in 3, probably representing small hot nodules, whereas normal thyroid was found in 9 patients. After the metastases to cervical lymph nodes was proven to be from the thyroid through lymph node FNA or lymph node dissection, all patients underwent total thyroidectomy, central department lymph node dissection and ipsilateral lymph node dissection. Moreover, in 3 patients lymph node dissection of the upper mediastinum was performed. At this point the patients were referred to the Endocrine clinic for further management.

Ablation therapy with a standard radioactive iodine^131 ^dose of 3700 MBq was given, followed by a whole body scan. In all patients suppressive thyroxine therapy at a dose of 1.5–2 μg/kg/day was given.

The second group included in the study (group B), comprised 30 consecutive patients (27 females) aged 28–70 years (mean 47.17 ± 13.17 years) who were similarly referred to the endocrine clinic by the surgery department after they had undergone total or near total thyroidectomy for multinodular goiter, and in whom pathological examination had revealed the incidental presence of a papillary microcarcinoma. The above patients underwent surgery, because one or more nodules had significantly increased in size during the last months; although they had no signs of malignancy in the ultrasound and the FNA was negative. During thyroidectomy inspection of lymph nodes status, both of central and lateral compartments was performed and it was negative for metastases. This second group served as the control group. At referral in the endocrine clinic, a neck ultrasound was performed which did not show the presence of metastatic lymph nodes. Thyroid bed uptake was under 1.5% in all patients. Thyroxine therapy at a dose of 1.5–2 μg/kg/day was given. No patient in this group received ablation therapy with radioactive ^131^I.

Postoperatively, in both groups, TSH and thyrogobulin (Tg) were measured after thyroxine withdrawal (table 1, 2).

**Table 1:** Surgery, pathology, and metastases in patients from group A

**Patient**	**Age**	**Tumor size (mm**)	**Pathology**	**Infiltration/Extension**	**Metastatic lymph nodes**	Tg **	Metastases *	**Follow up (mo)**	**Recurrence/persistent disease**
PA	50	10	P-f		5	27.6	Upper mediastinum Lymph nodes	39	CLN

TZ	43	4	P-f	Thyr capsule Thyr parenchyma	2	43.7		51	-

KΠ	58	8	P-f	-	1	59.3		41	-

GM	40	10 & 2 & 2	P-f	Thyr capsule Thyr parenchyma	4	0.4		36	-

MT	57	10 & 2 & 1	P-f	Thyr capsule Thyr parenchyma	7	43.0		75	CLN c

SE	37	4	P	-	11	1.9		60	-

KT	34	10 & 2	P	Thyr parenchyma Fatty tissue	3	19.5		140	CLN

KM	26	2 & 5	P	-	1	0.8		56	_

AA	56	5	P	-	1	25.7		34	CLN

LC	63	1	P-f	-	2	0.7		46	-

HD	33	8	P-f	Thyr capsule Thyr parenchyma	3	5.9		48	-

HS	56	10 & 3 & 2	P-f	Thyr parenchyma Fatty tissue	1	3.7		34	-

MD	38	7	P-f	-	1	7.7		44	-

TP	32	2 & 10	P	-	3	0.5		38	-

SP	37	8 & 10	P	Nodule capsule Fatty tissue	9	503.0	Lower mediastinum Lymph nodes	58	_

GK	24	7 & 8	P-f	-	2	2631.0	Parapharyngeal area	48	_

HI	28	9	P-f	-	1	5.3		36	-

ZS	57	10	P	Nodule capsule	2	8.7		40	-

KA	21	7	P	-	1	2.3		102	-

NG	16	9	P-f	Thyr capsule Thyr parenchyma	3	27.7	Upper mediastinum lymph nodes	56	CLN

PF	26	8 & 9	P-f	Thyr capsule Thyr parenchyma	8	134.0	Upper mediastinum lymph nodes	42	CLN

VA	58	8 & 5	P	Thyr parenchyma Fatty tissue	4	0.3		130	-

AS	42	8	P-f	Thyr capsule Thyr parenchyma	4	791.2		40	CLN

BE	27	5	P-f	Thyr capsule Thyr parenchyma	7	0.3		42	

P: papillary, P-f: Papillary-follicular type, Thyr: Thyroid, CLN: cervical lymph nodes, c: controlateral of initial disease, metastatic lymph nodes, *Postoperative thyroglobulin on stopping LT4, ** Distant metastases diagnosed with the post-therapy WBS

**Table 2:** Pathology of papillary thyroid microcarcinomas and Tg in group A and B

**Patients characteristics**	**Group A, n = 24**	**Group B, n = 30**	**P**
Mean age (years)	39.96 ± 13.91	47.17 ± 13.17	0.050
Mean tumor size * (mm)	7.71 ± 2.49	5.30 ± 2.67	0.001
% multifocality	10/24 (41.7%)	0/30 (0%)	0.000
% extrathyroid extension	4/24 (16.6%)	0/30 (0%)	0.000
% lymph nodes metastases	24/24 (100%)	0/30 (0%)	0.000
Tg **(ng/ml)			
range	0.3 – 2630	0.3 – 1.0	
median	8.2	0.75	

* In the cases with multifocal disease we have calculated only the maximum tumor size. ** Postoperative thyroglobulin on stopping LT4

### Pathology

All thyroid tissue samples were oriented, cut in parallel longitudinal slices 5 mm thick and fixed in 10% neutral buffered formalin for 24 hours. After fixation the samples were finely cut and paraffin embedded. For solitary encapsulated nodules measuring up to 5 cm, found only in group B, an additional section was taken for each additional centimeter in diameter, including the nodule capsule and adjacent thyroid tissue. For multinodular goiters, one section of each nodule (up to five nodules) was taken and more than one section for larger nodules [13]. Samples were routinely processed after paraffin-embedding. Normal tissue adjacent to neoplastic areas was also evaluated. The assessment of the diameter of the lesion was done microscopically. The tissue samples were all examined in the same Department by two pathologists who were consistent in looking for coincidental microcarcinomas. Pathologists were not aware of the clinical data.

### Statistical analysis

Statistical analysis of the differences between clinical and histological variables between groups was conducted by χ^2 ^test or Student's *t *test. Significant levels were presented as P values. Variables analyzed were: age, tumor size, number of tumor foci, lymph node metastases at thyroidectomy and invasion of thyroid parenchyma, thyroid capsule, extrathyroidal invasion or vessels invasion. Calculations were carried out using SPSS version 12.0. Results were considered statistically significant when p value was ≤ 0.05.

## Results

In all patients in group A, with impalpable thyroid gland and unknown thyroid disease, pathology confirmed the existence of papillary carcinoma of the thyroid, 1–10 mm in maximum diameter (mean 7.71 mm). In 10 cases (41.7%), more than one tumor foci were identified located in one lobe in 7 patients, and in both lobes in 3 patients. The carcinoma was entirely of the papillary type in 9 cases (37.5%), while in the remaining 15 cases was follicular variant of papillary thyroid carcinoma (Table 1). In all tumors, the carcinoma cells showed the characteristic nuclear morphology, with enlarged, crowded, clear nuclei (ground glass), often with longitudinal nuclear grooves. Psammoma bodies were found in 8 cases (33.3%). In 11 cases (45.8%), the tumor infiltrated the adjacent thyroid parenchyma, while in 8 (33.3%) it disrupted the thyroid capsule, and in 4 cases it extended into the surrounding adipose tissue (Table 1). In 16 patients (66.6%), many new metastatic cervical lymph nodes were found in both compartments. In most patients, lymph node metastases showed the typical papillary pattern, while in other cases mixed follicular-papillary morphology was also observed. The metastatic foci, in all cases, were of the same pattern with the primary tumor.

In all patients in group B pathology revealed unifocal papillary thyroid carcinoma of 1–10 mm in maximum diameter, (mean 5.3 mm). The carcinoma was entirely of the papillary type in 25 cases (83.3%), while in the remaining 5 cases it was follicular variant of papillary thyroid carcinoma. No infiltration to thyroid parenchyma and thyroid capsule or extension into the adjacent tissue, or cervical lymph node metastases were noted (Table 2). None of the larger nodules identified in this group was malignant, they all proved to be benign lesions.

**Figure 1:**
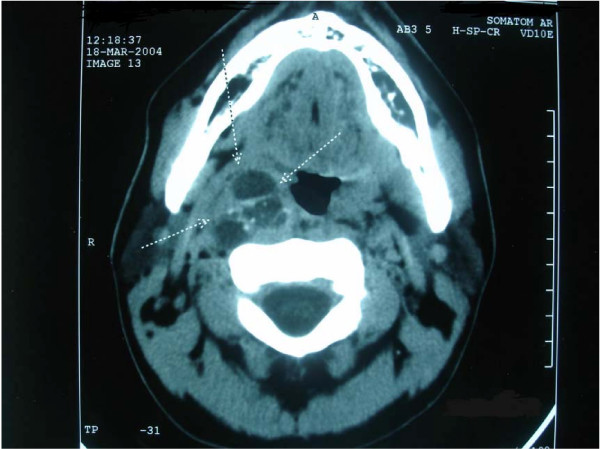
Computed tomography of a patient (GK) with metastasis in the parapharyngeal area from papillary thyroid microcarcinoma (for details see Table 1).

**Figure 2:**
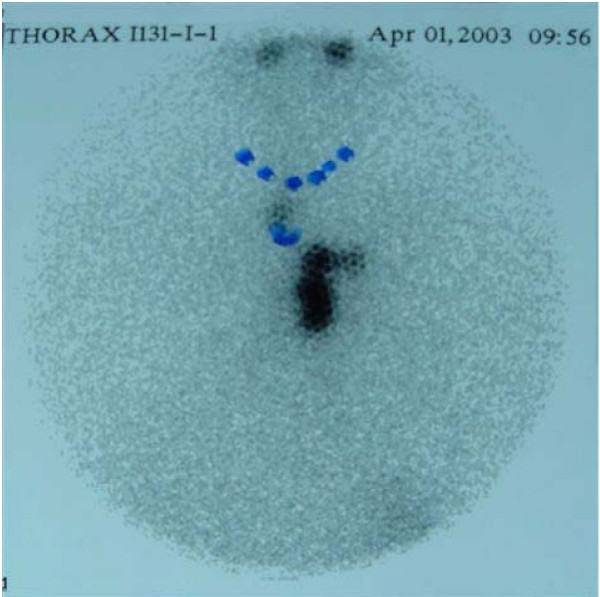
Post-therapy whole body scan of a patient (SP) with papillary thyroid microcarcinoma. Abnormal up take is observed in the mediastinum (for details see table 1).

In group A, serum thyroglobulin levels after thyroidectomy ranged from 0.3–2630 ng/ml, median 8.2 ng/ml, and TSH was 38.9–92.4 μU/ml (normal values 0.3–4 μU/ml) on stopping LT4. Higher levels of serum thyroglobulin appeared in patients with distant metastases (Table 1). A whole body scan, which was performed 7 days after ablation, revealed a large metastasis in the parapharyngeal area in 1 patient (Fig. 1) and in the mediastinum in another patient (Fig 2). Both patients underwent completion surgery. In group B, TSH was 36.5–79.8 μU/ml, and serum thyroglobulin ranged from 0.3 to 1.0 ng/ml, median 0.75 ng/ml after thyroxine withdrawal.

Statistical analyses between groups A and B revealed that mean age at the diagnosis of thyroid cancer was higher in patients of group B and this difference was borderline significant (t = -1.95, p = 0.05). Tumor size was significantly smaller in group B (t = 3.39, p = 0.001, table 2). Tg levels were higher in group A (table 2). Significant differences were found between group A and B when lymph node metastases, extrathyroid extension of the tumor and multifocality were compared (table 2).

### Follow-up

The follow-up duration of the patients in our clinic was 34–140 months (mean 55.66 months) in group A and 38–130 months (mean 62 months) in group B. All patients (in both groups) underwent clinical examination and an ultrasound scan of the neck every 6 months (group A) or 12 months (group B). Thyroid stimulating hormone, FT4, and Tg [14], on thyroxine or after stimulation with TSH [15] were measured annually; a whole body ^131^I scan was performed when necessary. Four patients in group A received additional radioactive iodine^131^I therapy (3700–7400 MBq) due to persistent local disease. During the follow up, and specifically 24–36 months after the initial treatment, 3 patients in group A presented with new metastases in the cervical lymph nodes (one contralateral of the initial disease) and received additional therapy with radioactive iodine [16] or underwent lymph node dissection [17]. During the last follow up, all patients were alive and free of disease. Patients in group B were also regularly followed up originally at 6 months and subsequently at 12 month intervals by detailed ultrasound and Tg measurements and none of them has so far presented with local or distant metastases.

## Discussion

Papillary thyroid microcarcinomas are small sized thyroid tumors that belong to the group of well-differentiated low risk carcinomas of the thyroid. They can be present in normal thyroids, just like in many of our patients. Many of them are tumors with benign behavior that do not affect patient survival and do not need supplementary radioactive therapy. As these tumors remain small or even regress, some authors suggest that incidentally found small papillary carcinomas should be called occult papillary tumors [18] instead of carcinomas. Similarly, a recent Annual Cancer Meeting held at the Institute of Molecular Pathology and Immunology of University of Porto, Porto, Portugal raised the issue of re-naming of papillary thyroid carcinomas, ≤ 1 cm in diameter (single focus), found incidentally within the thyroid gland of an adult undergoing thyroidectomy for some other reason, as "papillary microtumors" [19]. Occasionally, despite their small size, papillary thyroid tumors can give rise to cervical lymph node metastases and, less often, distant metastases [7,20]. Metastatic lymph nodes usually appear as a solid mass in the lateral neck. Cystic lymph node metastases are rare [21,22]. Two of our patients presented with a lateral cystic cervical mass, resembling a branchial cyst. A preoperative diagnosis between a branchial cyst and a solitary cystic lymph node metastasis from occult thyroid carcinoma may be difficult [23,24]. It has been suggested that thyroglobulin measurements can be performed from the aspirate of the cystic lesion and if this is measurable, then a metastasis from a thyroid carcinoma is very likely [25]. Unfortunately this method was not applied in our study. It should be stressed that lateral cervical masses may be of thyroidal origin even in the absence of evident thyroid disease [26] as was observed in three of our patients in group A, who had very small tumors with a maximum diameter of 1, 4 and 4 mm. This concept is not always obvious and can lead to misdiagnosis or delayed diagnosis and to unnecessary 2 step surgeries.

Although the risk of distant spread is certainly lower for small tumors, sporadic cases of "occult" tumors with distant metastases have been reported [7,27,28]. Two of our patients had distant metastases at diagnosis of the disease (one in the parapharyngeal area and one in the lower mediastinum, which were both surgically removed). It should be noted that the absence of suspect clinical findings in the thyroid does not exclude local or distant metastases from small thyroid cancers, as was indeed observed in our patients

Group A patients with small thyroid tumors did not differ in their clinical behavior from patients with larger papillary carcinomas of the thyroid. Therefore, we believe that criteria other than size alone should be taken into consideration when observing the clinical behavior of thyroid tumors [6,11,12]. The histological findings distinguishing invasiveness in lymph and blood vessels, thyroidal capsular invasion or extrathyroidal extension, low differentiation of tumor cells, and multifocality are critical and may influence therapeutic decisions and follow up. Most patients in group A had such histological findings. Moreover in our study, a difference in papillary thyroid cancer variants was observed between groups. The carcinoma was entirely of the papillary type in 37.5% of patients in group A and in 83.3% of group B; follicular variant of papillary thyroid carcinoma was found in 62.5% and 16.7% respectively. Consequently, we suggest that not all small carcinomas should be grouped together based on size alone, and the existence of two biological groups of "apparently occult" papillary thyroid carcinomas, characterized by a very different clinical behaviour, must be considered; the clinically apparent thyroid microcarcinoma and the incidentally found thyroid carcinoma. The presence of lymph-nodes is obviously taken into account in the classification and the therapy planning of thyroid tumours as has recently been suggested in the recommendations of both the American and the European Thyroid Associations [16,29]. True incidental occult papillary carcinoma seemed to be different from non-incidental or clinically apparent papillary microcarcinoma regarding the main prognostic features [10,30-32]

Recent publications reveal that papillary microcarcinomas with preoperatively detectable lymph node metastases present with significantly more aggressive characteristics than those with no metastases [33]. Four of our patients in group A received additional radioactive iodine^131^I therapy due to persistent local disease and 3 of the same group presented with new cervical lymph nodes metastases (one contralateral of the initial disease) 24–36 months after the initial treatment, and received additional therapy with radioactive iodine or underwent lymph node dissection. Ito, in a recent publication [33], immunohistochemically examined the expression of cell proliferation markers and apoptotic markers in microcarcinoma patients with and without clinically apparent metastases, and found that cases with clinically apparent metastases showed increased cyclin D1 expression together with decreased p27 expression and higher levels of pRb and Ki-67 expression. These findings suggest that cases with clinically apparent metastases show significantly higher growth based on cell proliferation activity, apoptosis, and expression of metastatic suppressors than those demonstrating no metastases.

Considering that some very small papillary thyroid carcinomas may give rise to recurrences and local or distant metastases, we regard that their therapy should not be different than that reported for the larger ones, especially if cervical lymph node metastasis was the reason for surgery. In other words, total thyroidectomy and lymph node dissection [34-39] should be followed by suppressive thyroxine therapy [40] and radioactive iodine ablation therapy [41-43]. In cases of aggressive disease, the bilateral and multifocal nature of otherwise occult primary disease argues for total thyroidectomy. Ten of our patients of group A had multifocal disease (in three of them the disease was bilateral). As most of the recurrences are in the thyroid and the neck region, careful neck palpation supplemented with high-resolution ultrasonography represents a useful surveillance strategy. During surgery, special care should be given to the central lymph node compartment, where the majority of lymph node metastases from PTC has been reported [39,44,45]. Prophylactic lymph node dissection of the central compartment should be considered in patients who undergo thyroidectomy for microcarcinomas and who present lateral lymph node metastasis which are either known preoperatively by ultrasonography or which are palpable during surgery [9]. Prophylactic node dissection was not shown to be beneficial in patients without palpable lymphadenopathy, as the recurrence rate did not differ between the prophylactic lymph node dissection group (0.43%) and the no-dissection group (0.65%) [46]. It should be noted however, that despite the local invasiveness and distant metastases of these small carcinomas, the reported death is extremely rare [47]. All our patients are still alive.

The majority of our patients of group B did not undergo central lymph node dissection. However, during follow up (range 38–130 months, median 106 months) no recurrence in cervical lymph nodes or distant metastases appeared. In a recent publication, the authors present the results of 317 patients who underwent surgery for benign diseases and were found to have a papillary thyroid microcarcinoma undetected by preoperative ultrasonography and fine-needle aspiration biopsy. After the diagnosis of incidental papillary thyroid carcinoma none of the patients underwent a completion central lymph node dissection. During follow up only 2.2% presented recurrent disease [45].

From this retrospective study, we conclude that papillary microcarcinomas can be diagnosed after they have produced cervical lymph node metastases which may be the first manifestation of the disease. Clinically apparent lymph node metastasis is predictive of multifocality, higher probability of extrathyroidal extension and a tenedency of recurrence. Therefore, when a lateral cervical mass is present, despite the fact that the thyroid gland may seem normal, a small thyroid carcinoma should be considered as likely. In the case of lesions that are suspicious to be thyroid-derived, FNA of the lateral mass is usually diagnostic, and is preferable to an open biopsy. Small tumor size alone is not a reliable prognostic marker, and treatment planning should be guided by histological findings such as limitation of the tumor, invasiveness, or lymph node metastases.

## Competing interests

The authors declare that they have no competing interests.

## Authors' contributions

KPP conceived the research work, coordinated the data collection and prepared the manuscript. MA contributed to the revision of the manuscript and performed the statistical analysis. MB reviewed the patients records. AD reviewed the patients records. AK examined tissue samples. IV coordinated the study. All authors read and approved the final manuscript.
